# Rosmarinic Acid-Loaded Polymeric Nanoparticles Prepared by Low-Energy Nano-Emulsion Templating: Formulation, Biophysical Characterization, and In Vitro Studies †

**DOI:** 10.3390/ma15134572

**Published:** 2022-06-29

**Authors:** Jessica García-Melero, Joan-Josep López-Mitjavila, María José García-Celma, Carlos Rodriguez-Abreu, Santiago Grijalvo

**Affiliations:** 1 Institute for Advanced Chemistry of Catalonia (CSIC-IQAC), Jordi Girona 18-26, E-08034 Barcelona, Spain; jessygarcia.melero@gmail.com (J.G.-M.); joanlopezmitjavila@outlook.es (J.-J.L.-M.); 2 Department of Pharmacy, Pharmaceutical Technology, and Physical-Chemistry, R+D Associated Unit to CSIC Pharmaceutical Nanotechnology, IN2UB, University of Barcelona, Joan XXIII 27-31, E-08028 Barcelona, Spain; mjgarcia@ub.edu; 3 Networking Centre in Bioengineering, Biomaterials and Nanomedicine (CIBER-BBN), Jordi Girona 18-26, E-08034 Barcelona, Spain

**Keywords:** antioxidant activity, cellular uptake, colloidal dispersions, drug delivery, nano-emulsions, nanoparticles, phytochemical, PLGA, protein corona

## Abstract

Rosmarinic acid (RA), a caffeic acid derivative, has been loaded in polymeric nanoparticles made up of poly(lactic-*co*-glycolic acid) (PLGA) through a nano-emulsion templating process using the phase-inversion composition (PIC) method at room temperature. The obtained RA-loaded nanoparticles (NPs) were colloidally stable exhibiting average diameters in the range of 70–100 nm. RA was entrapped within the PLGA polymeric network with high encapsulation efficiencies and nanoparticles were able to release RA in a rate-controlled manner. A first-order equation model fitted our experimental data and confirmed the prevalence of diffusion mechanisms. Protein corona formation on the surface of NPs was assessed upon incubation with serum proteins. Protein adsorption induced an increase in the hydrodynamic diameter and a slight shift towards more negative surface charges of the NPs. The radical scavenging activity of RA-loaded NPs was also studied using the DPPH·assay and showed a dose–response relationship between the NPs concentration and DPPH inhibition. Finally, RA-loaded NPs did not affect the cellular proliferation of the human neuroblastoma SH-SY5Y cell line and promoted efficient cellular uptake. These results are promising for expanding the use of O/W nano-emulsions in biomedical applications.

## 1. Introduction

Phytochemicals constitute a myriad of chemical structures including phenolic acids, alkaloids, flavonoids, or indoles, among many others [1,2]. These small molecules in their native or in secondary metabolite form have drawn interest in therapy owing to various pharmacological properties such as antioxidant activity, free radical scavenging, or metal chelation [3]. These features have contributed greatly to potentiating the benefits of phytochemicals for human health and management of disease prevention [4].

Rosmarinic acid (RA) is a naturally occurring caffeic acid ester containing a 3,4-dihydroxyphenylacetic acid moiety. RA itself exhibits antiviral, anti-inflammatory, and antioxidant activities [5,6,7]. RA and many other phytochemicals are expected to maintain homeostasis and mitochondrial function which have turned into promising neuroprotective agents [8]. For instance, RA has shown its effectiveness in reducing Aβ amyloid aggregates by increasing monoamine secretion and suppressing tau phosphorylation in animal models [9,10]. These remarkable properties have made RA a promising molecule to prevent neurodegeneration and improve brain dysfunction [11].

Some recent in vitro strategies have contributed to detecting flavonoids in neuroblastoma cells using fluorescence enhancers [12]. However, these natural products including other phytochemicals exhibit low absorption rates and are prone to be metabolized in vivo giving rise to rapid clearance from the bloodstream [13]. As a result of these limited pharmacokinetic properties, the accumulation of phytochemicals within specific target tissues including brain is quite limited [14]. To protect phytochemicals from enzymatic degradation and thus enhance their circulation time, nanotechnology-based strategies represent a promising approach aimed at increasing drug bioavailability, enhancing their safety profiles, and favouring drug delivery and drug permeation into target cells and tissues [15].

Further advances in controlling nanoparticles’ (NPs) architecture and the use of conjugation strategies to decorate NPs’ surfaces with specific targeting ligands have accelerated the inclusion of these nanomaterials from the bench toward clinical applications [16]. In this sense, the use of biodegradable polymers has attracted much attention in engineering a vast number of polymeric NPs with long lifetimes, including the ability to deliver a myriad of entrapped cargoes. These features make polymers appropriate materials for diverse bio-applications [17,18,19]. To this end, polymeric NPs made up of poly(lactic-*co*-glycolic acid) (PLGA) have been widely used in biomedical research owing to their biocompatible and biodegradable properties [20] necessary to transport not only small molecule drugs but also peptides and macromolecules [21,22,23]. Interestingly, PLGA degradation rates can also be modulated depending on both molecular weight and the copolymer ratio. However, the accumulation of the resultant polymer degradation compounds at high concentrations has shown certain cytotoxicity in several cell culture lines and has been related to the osmolality of the culture medium [24].

Nano-emulsions (NEs) are nano-sized non-equilibrium liquid-liquid dispersions. NEs can be obtained either by high-energy emulsification methods or low-energy methods. While high-energy methods require the use of mechanical devices [25], low-energy emulsification methods take advantage of the internal energy present in the system and rely on changes in the spontaneous curvature of the surfactant layer surrounding the emulsion droplets. Such changes can be triggered by varying temperatures as in the phase-inversion temperature (PIT) method or composition as in the phase-inversion composition (PIC) method [26,27]. Low-energy methods also have advantages such as generating smaller droplet sizes, easiness to scale up, and the possibility of processing at room temperature [28,29,30].

NEs are a versatile tool to engineer drug delivery systems in the form of polymeric NPs [31,32]. In this regard, it is expected that NPs may enhance the pharmacological effect of drugs when compared with the drug in the free form [33]. Previous research carried out by our group involved the preparation of O/W polymeric nano-emulsion templates using the PIC method and a ternary system consisting of three pseudo-components: an aqueous solution, a solution of PLGA in ethyl acetate and a surfactant (i.e., Tween-80^®^). As a result, stable polymeric NPs with well-defined and precise size control were obtained after ethyl acetate evaporation from the NE. Interestingly, the resultant NPs were shown to be effective not only in promoting cellular uptake of macromolecules [34] but also in facilitating the transport of small molecules across the blood–brain barrier (BBB) [35].

One of the limiting factors that involves the use of NPs in vivo and their final process for clinical translation, is their interaction with proteins present in the bloodstream [36]. Proteins are spontaneously adsorbed onto the NPs’ surface leading to the formation of a protein corona (PC) [37]. The biophysical characterization and biological activities of PC have been widely studied in the case of liposomes or metallic NPs but scarcely reported for polymeric NPs [38,39]. The effect of PC on the NPs’ biological fate is still controversial [40] and has shown a direct impact on NPs toxicity, cell internalization, or drug cargo release, among others [38]. Despite the fact that these parameters can inevitably reduce the biological activity owing to the coating of non-specific proteins bound to the NPs’ surface, the presence of other proteins such as albumin and apolipoproteins may contribute to the opposite effect [37,41]. Therefore, analyzing and characterizing the PC layer is a key step toward developing polymeric delivery systems.

Our group has devoted much effort to using low-energy emulsification methods, in particular the PIC method, as a versatile approach for the preparation of drug-loaded polymeric NPs from nano-emulsion templates. Thus, this strategy has allowed us to consider alternate therapeutic strategies based on polymeric NPs aimed to transport specific drugs toward the central nervous system for the first time [35]. Our findings suggested that PLGA NPs were able to pass through the BBB. These outcomes prompted us to search for potential strategies to deliver antioxidant agents aimed to reduce cellular oxidative stress. Inspired by the promising activities of certain phytochemicals in the field of neurodegenerative diseases, herein we report the preparation of RA-loaded polymeric NPs from NEs with the aim to find suitable formulated systems to administer RA (Figure S1). Additionally, various studies including biophysical analysis, controlled delivery of RA, the effect of the protein corona (PC) on the colloidal stability, antioxidant activity, as well as in vitro cytotoxicity, and cellular uptake of RA-loaded PLGA NPs have been carried out using SH-SY5Y as a neuronal model cell line [42]. These results are aimed at gaining insight into further understanding of NPs behaviour, particularly those aspects related to their use in the treatment of neurological diseases.

## 2. Materials and Methods

General information. All solvents and chemical reagents were of reagent grade and used as received. PLGA (Resomer 752H, MW ~ 10,000 g/mol from Boehringer Ingelheim, Ingelheim am Rhein, Germany) was used as a biocompatible and biodegradable polymer for the preparation of NPs. Rosmarinic acid (RA) at 97% purity was purchased from Alfa Aesar (Haverhill, MA, USA). Ethyl acetate and ethanol were purchased from Merck (St. Louis, MO, USA). Polysorbate 80 was purchased from Croda (Snaith, UK). Sodium chloride (NaCl) (Carlo Erba, Catalonia, Spain), potassium chloride (KCl) (Merck, St. Louis, MO, USA), disodium monohydrogenphosphate dihydrate (Na_2_HPO_4_·H_2_O) (Merck, St. Louis, MO, USA), potassium dihydrogen phosphate (KH_2_PO_4_) (Merck, St. Louis, MO, USA) and Milli-Q ultrapure water (Merck Millipore, Barcelona, Spain) were used to prepare phosphate-buffered saline (PBS, pH 7.4, 300 mOsm/kg). Acetonitrile, HPLC grade was purchased from Fisher Scientific (Waltham, MA, USA). The dialysis membrane Spectra/Por was purchased from Repligen Corporation (Waltham, MA, USA). Fetal bovine serum (FBS) (Thermofisher Scientific, Waltham, MA, USA) was used as a growth supplement for cell culture media. Dubelcco’s Modified Eagle Medium (DMEM), Trypsin–EDTA (0.05%) was purchased from Gibco (Thermofisher Scientific, Waltham, MA, USA). A human cell line SH-SY5Y was provided by the Cryogenics core facility of the University of Barcelona. 3-(4,5-dimethylthiazol-2-yl)-2,5-diphenyltetrazolium bromide (MTT) (Merck, St. Louis, MO, USA) and dimethyl sulfoxide (DMSO) (Panreac, Barcelona, Spain) were used to test cytotoxicity. Bicinchoninic acid and copper (II) sulphate solution (Merck, St. Louis, MO, USA) were used as reagents for the BCA assay.

### 2.1. Preparation of Polymeric Nano-Emulsions

Non-loaded and drug-loaded polymeric NEs were prepared using the PIC method [43] at room temperature. O/W polymeric NEs were prepared by adding 1× PBS (3.6 mL, 90%) dropwise at a constant flow rate to mixtures made up of surfactant Polysorbate 80 (0.120 mL, 3 wt%) and 4 wt% PLGA in ethyl acetate (0.280 mL, 7 wt%). RA-loaded NEs were prepared using the same protocol but adding RA in the organic phase at different concentrations. Finally, translucent O/W NEs were obtained [35].

### 2.2. Preparation of Polymeric Nanoparticles from Nano-Emulsion Templating

Polymeric NPs were obtained from their NE counterparts by the solvent evaporation method. The evaporation conditions were carried out in a Büchi R-215V Rotavapor (Flawil, Switzerland ) at 25 °C, with a rotation speed of 150 rpm and a vacuum of 43 mbars for 50 min. After the evaporation was completed, the final volume of NPs was adjusted with Milli-Q water to maintain the osmolality of the sample at around 300 mOsm/kg. For RA-loaded PLGA NPs, an additional dialysis step using 1× PBS as a receptor phase was performed to purify the polymeric NPs by removing the amount of RA that was not entrapped.

### 2.3. Physicochemical Characterization of Nanoparticles

The mean droplet size and size distribution of polymeric NPs were determined by dynamic light scattering (DLS). A spectrometer (LS instrument, Fribourg, Switzerland, 3D cross-correlation multiple scattering) equipped with a He-Ne laser (632.8 nm) at a scattering angle of 90° and at 25 °C was used. The correlation functions of scattering data were analyzed by the cumulants method, and the hydrodynamic size was calculated by the Stokes-Einstein equation assuming a spherical shape of the nanoparticles. DLS results represent the average of three measurements for each sample. The zeta potential of polymeric NPs was assessed by electrophoretic mobility measurements with a Zetasizer NanoZS instrument (Malvern Instruments, Malvern, UK), equipped with a He-Ne laser (633 nm). The zeta potential (ζ) was calculated according to the Hückel–Onsager Equation (1).

(1) $$\zeta \;=\frac{2\cdot \eta \cdot \mu }{3\cdot \varepsilon_{r}\cdot \varepsilon_{0}}$$

where, ε_r_ is the relative dielectric constant of water, ε_0_ is the vacuum permittivity, μ is the electrophoretic mobility and η is the liquid viscosity. Additionally, the stability of RA-loaded NPs over time was performed by monitoring their average diameter size for one month at room temperature according to the DLS protocol described above.

### 2.4. Rosmarinic Acid (Ra) Quantification Assays

A Breeze™2 HPLC instrument (Waters Corporation, Milford, MA, USA) equipped with a Spherisorb^®^ 5 µm 4.6 × 150 mm analytical column was used to analyze RA concentration at 328 nm. The mobile phase used was Milli-Q water (solvent A) and acetonitrile (solvent B). Gradient elution conditions were applied (Table S1) injecting 50 µL of the final sample with a flow rate of 1.00 mL/min. Each chromatogram was plotted for 20 min. The retention time of RA for this gradient method was ca. 6.2 min.

### 2.5. Encapsulation Efficiency (EE%)

The encapsulation efficiency of RA was determined by centrifuging the resultant RA-loaded NPs using centrifugal filter units with a molecular weight cut-off of 3 KDa (Atomic Ultra-15 Centrifugal Filter Unit with Ultracell-3 Membrane, Millipore, Billerica, MA, USA). The centrifugation process was carried out using 1.00 mL of the RA-loaded NPs dispersion at 4 °C for 75 min. The filtrate was analyzed by HPLC to quantify the amount of RA that was not encapsulated. A calibration curve was obtained from several RA concentrations ranging from 1000 to 31.2 µg·mL^−1^. The encapsulation efficiency (EE%) was calculated using (2).

(2) $$\mathrm{EE}\;\left(\%\right)=\;\frac{total\;drug\;amount-free\;drug}{total\;drug\;amount}\times 100$$

### 2.6. Hyperspectral Microscopy and Dark-Field Imaging

Hyperspectral images of RA-loaded PLGA NPs were visualized using hyperspectral enhanced dark-field microscopy. This system consists of an Olympus BX-43 microscope coupled to a Cytoviva© (Auburn, AL, USA) high-resolution dark-field condenser and spectral imaging system. The hyperspectral camera system operates in the visible-near infrared (VNIR) range (400–1000 nm). Microscope images were acquired using Exponent 7 software and the corresponding hyperspectral images were collected using ENVI software. Firstly, RA and PLGA solutions were scanned to obtain a library of hyperspectral images which acted as a reference. RA-loaded NPs (5 µL) were scanned, and the resultant hyperspectral images were compared with the reference spectra previously obtained.

### 2.7. Transmission Electron Microscopy (TEM)

The morphology of RA-loaded PLGA NPs and PC NPs was assessed by transmission electron microscopy (TEM). Briefly, 5 μL of the sample was adhered onto glow discharged carbon-coated grids for 60 s. The remaining liquid was removed using a cellulose filter paper and was stained with 2 wt% phosphotungstic acid for 60 s. A Jeol JEM1010 transmission electron microscope (Tokyo, Japan) was used to visualize the samples using an accelerating voltage of 200 KeV in a bright-field image mode. Digital images were acquired using a digital camera (Olympus SIS Morada).

### 2.8. In Vitro Drug Release Experiments

The release of RA from polymeric NPs (2.0 mL) was studied in vitro over time. RA (2.4 mg; 6.6 µmol) was encapsulated following the experimental protocol described before. Release studies were carried out using the dialysis bag method at a pH of 7.4. This dialysis membrane is composed of regenerated cellulose with an MWCO of 3000 Da. Dialysis bags were pre-wetted in the presence of a 1× PBS solution for 10 min. Experiments were performed in triplicate using 30 mL of 1× PBS as a receptor solution at 37 °C, maintaining sink conditions. Aliquots of 1 mL of the receptor solution were withdrawn at appropriate intervals of time and the RA concentration was finally analyzed by HPLC. The diffusion of RA from an aqueous solution (2.4 mg of RA in 2 mL of PBS) was monitored in triplicate as control.

### 2.9. Mathematical Models

Different kinetic models were fitted to cumulative RA released as a function of time. Four drug release mathematical models were used: (i) zero-order (3); (ii) first-order (4); (iii) Higuchi (5) [44] and (iv) Korsmeyer–Peppas (6) [45]. M_∞_ and M_t_ represent the maximum and cumulative amounts of drug released at time “*t*”, Q_0_ is the initial amount of drug, and K (K_0_, K_H_, or K_K−P_) is a constant value that provides information regarding the geometric and structural properties of the dosage form. Finally, *n* is the release or diffusional exponent. When *n* is around 0.5 (Case I), a Fickian mechanism is considered whereas an anomalous diffusion is produced when 0.5 < *n* < 1 (Case II or non-Fickian). Drug release kinetics were obtained by fitting the corresponding RA release curve to the equation release models described below using DDSolver software [46].

(3) $$\frac{M_{t}}{M_{\infty }}=Q_{0}+K_{0}*t$$

(4) $$\frac{M_{t}}{M_{\infty }}=100*\left(1-e^{-K*t}\right)$$

(5) $$\frac{M_{t}}{M_{\infty }}=K_{H}*\sqrt{t}$$

(6) $$\frac{M_{t}}{M_{\infty }}=K_{K-P}*t^{n}$$

### 2.10. Protein Corona Formation

The formation and quantification of PC adsorbed on the PLGA NPs’ surface were carried out in fetal bovine serum (FBS). The assay was performed using bare polymeric NPs as a control, and RA-loaded NPs. A dispersion of NPs (500 µg/mL) in 1× PBS was mixed with 10% FBS to get a final volume of 2.0 mL. The mixture was incubated for two periods of time (1 and 5 h) at 37 °C. At the end of each incubation time, samples were ultracentrifuged (15 min; 15,000 rpm; 4 °C) to eliminate those proteins that were not bound to NPs. Finally, the resultant pellet was resuspended in 2 mL of PBS and PC_NPs were characterized by DLS and zeta potential. The protein content was quantified using the bicinchoninic acid (BCA) assay according to the manufacturer’s procedure. The protein content was measured at 562 nm. All experiments were carried out in triplicate.

### 2.11. In Vitro Antioxidative Activity and EC_50_

Radical scavenging activities of RA-loaded PLGA NPs, PLGA NPs, and free RA were studied using the 2,2-diphenyl-1-picryl-hydrazyl-hydrate (DPPH·) assay. The measures were made in triplicate adding the appropriate amount of the sample to a 96-well plate and adjusting the final volume to 220 µL with a DPPH· solution of 0.2 mM. Samples were incubated for 30 min in the darkness at room temperature and the absorbance was measured at 517 nm with the SYNERGY H1 spectrophotometer (BioTeck) (Agilent Technologies) (Santa Clara, CA, USA). A control sample was prepared with 20 µL ethanol and 200 µL of the 0.2 mM DPPH· solution. The antioxidant activity of non-loaded NPs, RA- loaded NPs and free RA was evaluated at 0.112 mg·mL^−1^ according to (7) [47]. Various concentrations of free RA and RA-loaded PLGA NPs were prepared to determine the EC50. Specifically, eight concentrations were prepared, starting from a solution of 0.16 mg·mL^−1^, taking half of the previous concentration (500 µL) and diluting until 1.0 mL, being 0.0013 mg·mL^−1^ as the last concentration. The EC_50_ corresponds to the concentration for which the inhibitory activity is equal to 50%.

(7) $$Scavenging\;activity\;\left(\%\right)=\left(1-\frac{A_{sample,\;517\;\mathrm{nm}}}{A_{control,\;517\;\mathrm{nm}}}\right)\times 100$$

### 2.12. In Vitro Cytotoxicity Test

Cell viability was assessed with the 3-(4,5-dimethylthiazol-2-yl)-2,5-diphenyltetrazolium bromide (MTT) colourimetric assay. For each assay, SH-SY5Y cells were seeded (6·10^3^ cells per well) on a 96-well plate in 200 µL of DMEM and cultured for 24 h at 37 °C under a 5% CO_2_ atmosphere. The culture medium was replaced with samples (bare PLGA, RA-loaded PLGA NPs, and free RA) prepared at appropriate concentrations (0.47, 0.23, and 0.112 mg·mL^−1^) to get a final volume of 200 µL. PLGA NPs and SH-SY5Y cells were incubated for 24 h at 37 °C. DMEM was discharged and replaced with fresh medium (200 µL). Cells were additionally incubated for 15 h. The MTT reagent was added at a final concentration of 0.5 mg/mL (25 µL) in PBS and incubated for 2 h at 37 °C. The medium was withdrawn and 200 µL of DMSO was added to dissolve the formazan crystals. The plate was shaken for 15 min at room temperature to facilitate the dissolution of the formazan crystals. Finally, absorbance was measured at 570 nm with a SpectraMax M5 spectrophotometer (Molecular Devices) (San Jose, CA, USA). Cytotoxicity experiments were performed in triplicate and cellular viability was calculated as a percentage compared to untreated control cells using (8).

(8) $$\%\;cell\;viability=\frac{Absorbance\;of\;treated\;cells}{Absorbance\;of\;control\;cells}\times 100$$

### 2.13. Flow Cytometry

Firstly, fluorescently labelled polymeric NPs were obtained as described above but loading Cou-6 (0.01 mg/mL) within the PLGA polymeric matrix. SH-SY5Y cells (300 × 10^3^ cells/well) were seeded on a 24-well cell culture plate and incubated for 24 h at 37 °C. Cou-loaded PLGA (0.23 mg/mL of PLGA) was mixed with cells and the volume was adjusted to 300 µL with DMEM. Cells were analyzed after 24 h incubation. Cells were washed with PBS (2 × 300 mL) and incubated with trypsin–EDTA (200 mL) at 37 °C for 5 min. DMEM (600 mL) was added, and cells were centrifuged (12.3 rpm for 5 min). Finally, pellets were analyzed by a flow cytometer instrument (Guava Incyte) (MilliporeSigma) (Burlington, MA, USA). For each sample, a selected gate (R1) was used, and 10^4^ events were collected that correspond to the SH-SY5Y cell population. A Flowing Software 2.5.1 (University of Turku, Finland) was used to analyze the ratio between untreated cell populations and fluorescently labelled cells.

Statistical analysis. Data are expressed as the mean ± SD. A two-sided Student’s test was used to analyze the differences between two groups. Differences were statistically significant when the *p* value was less than 0.05 (** *p* < 0.05).

## 3. Results

### 3.1. Preparation of Polymeric Nanoparticles

A NE containing 1× PBS (90%), 4% PLGA in ethyl acetate (EtOAc) (7%) and Polysorbate 80 (3%), which was used as a template for the preparation of polymeric NPs, was prepared using a low-energy emulsification PIC method [34,35,41]. As a result, the corresponding unloaded PLGA polymeric NPs with average sizes of 46.6 nm and PDI values of 0.29 were obtained upon evaporation (Figure S2) [34,35,41]. Likewise, RA-loaded NPs were prepared following the same protocol but dissolving specific amounts of RA in the organic phase using either EtOAc exclusively or a mixture of ethanol (EtOH) and EtOAc in a ratio of 10:90 (Table 1).

**Figure 1:**
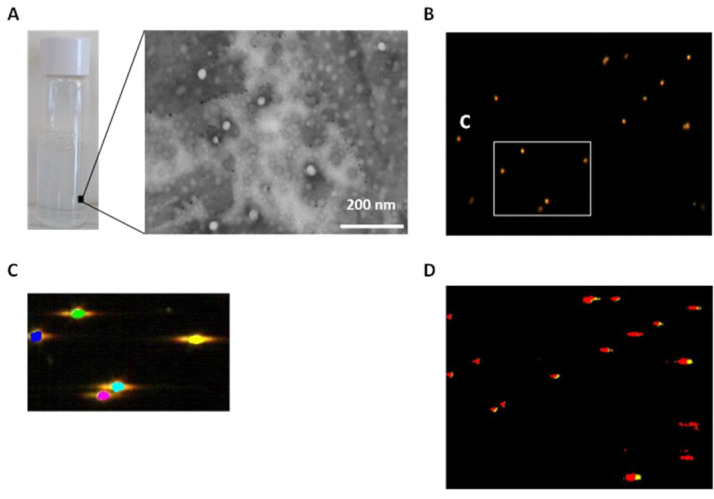
(**A**): Biophysical characterization of RA-loaded PLGA NPs: (**A**): Physical appearance and TEM microscopy image. Scale bar = 200 nm; (**B**): Dark-field microscopy image at 100× magnification; (**C**): Dark-field hyperspectral image selection; (**D**): Mapped region of RA-loaded PLGA NPs. Colour code for RA-loaded PLGA NPs: PLGA (red) and RA (yellow). The scattering spectra of PLGA and RA-loaded PLGA NPs are displayed in Figure S4.

Two RA concentrations (0.3 and 0.4 mg·mL^−1^) were selected to entrap RA within the PLGA matrix. In addition, two strategies involving either vortex (A) or combining vortex and a sonication process (B) were used. Data showed that polymeric NPs prepared in both cases exhibited average sizes ranging from 70 to 168 nm and PDI values of 0.2–0.3 (Figure S3). This increase in size might be attributed to the presence of the drug within the core of NPs (Figure 1A). Curiously, diameter sizes of NPs prepared in the presence of EtOH were larger than those prepared in only EtOAc but with similar nanoparticle size distribution in both cases. Thus, ethanol might affect the initial nano-emulsion formulation by changing surfactant-medium interactions and interfacial tensions. Therefore, larger droplets and NP sizes were obtained. All NPs dispersions showed negative ζ-potential values owing to the presence of free carboxyl groups in the PLGA polymer that impart a negative charge to the surface in an aqueous solution [48]. In addition, these negative values slightly increased (in absolute value) as the amount of RA increased ranging from −21.9 to −35.5 mV. These negative values are considered to provide enough electrostatic repulsion between NPs resulting in high colloidal stability and avoiding undesirable aggregation in vivo [49]. We also noted that the EE% dropped from 92% to 64% if RA content was increased from 1.2 to 1.6 mg. This significant fall in EE% has been also observed when entrapping other small molecules [50] and might be attributed to the partitioning of the drug into the aqueous phase. Thus, the presence of ethanol might increase RA diffusion from the NPs to the bulk solution leading to a fall in the EE% as detailed above. Previous findings have shown that increasing PLGA concentration might result in a higher entrapment efficiency but also lead to larger particle sizes [51]. Therefore, we selected the lowest concentration of RA (0.3 mg·mL^−1^) for further in vitro experiments. TEM microscopy images of our RA-loaded polymeric NPs revealed the spherical shape and showed similar diameter sizes as those obtained from DLS measurements (Figure 1A). Additionally, a dark-field image at 100× magnification and a dark-field hyperspectral image [52] of RA-loaded PLGA NPs were used to confirm the presence of RA entrapped in PLGA NPs (Figure 1B,C). For that purpose, the scattering spectra from the dark-field image of each component (e.g., RA and PLGA) were separately obtained (Figure S4) allowing the spectral image of RA-loaded PLGA NPs to be mapped (Figure 1D).

### 3.2. In Vitro Drug Release

**Figure 2:**
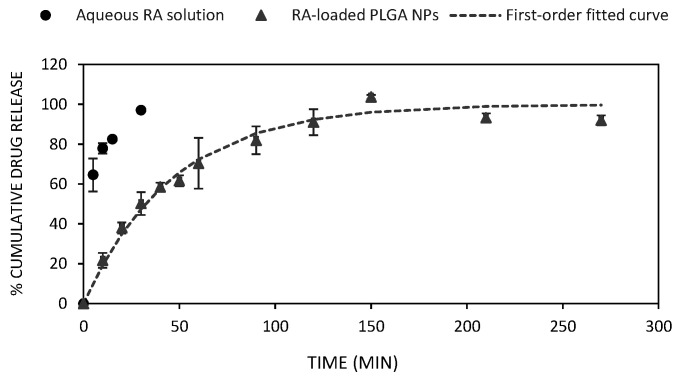
Drug release profile of RA-loaded PLGA NPs (grey dots) under physiological conditions (pH 7.4, 37 °C) using 1× PBS as receptor solution. The diffusion of free RA (black dots) was studied as a control experiment. First-order kinetic model (*r*^2^ = 0.992) fitted cumulative percentage of RA released from PLGA NPs (dashed line). Data correspond to the mean of three independent experiments.

The in vitro drug release evaluation of RA-loaded PLGA NPs was carried out by the dialysis method in 1× PBS at physiological conditions (pH 7.4 and 37 °C) under sink conditions and their cumulative percentage release over time is summarized in Figure 2. The diffusion of RA from a solution was determined for comparative purposes.

The drug release diffusion from the nanocarrier through the dialysis membrane to the receiver solution was monitored by HPLC at 328 nm at different intervals of time. Therefore, the cumulative amount released of RA at each time point was calculated from the corresponding standard curve (Figure S5). On the whole, the diffusion rate of free RA (black dots, control) was much faster if compared with RA-loaded PLGA NPs (grey dots) which afforded a cumulative release of 93% of the total loaded RA reaching a plateau at 270 min (Figure 2). While the first 30 min afforded a complete diffusion of RA (97%) in an aqueous solution, only 46% of the drug was released from the polymeric NPs at the same experimental time. This sign of delayed cumulative release may clearly support the adequate encapsulation of RA within the polymeric NPs [35]. The initial burst release observed (21% in the first 10 min) might be attributed to the presence of RA in the outer layer of the NPs whereas the diffusion of RA from the PLGA interior layers might corroborate the subsequent sustained release behaviour of the system [53].

Various mathematical models estimate the drug release kinetics from nanocarriers [54,55]. Herein, four models (e.g., zero-order, first-order, Higuchi and Korsmeyer–Peppas) have been selected to elucidate and gain insight not only into the diffusion but also the physical and chemical mechanisms that govern the release of RA from PLGA NPs. The values for each parameter of the selected models are summarized in Table S2. In terms of *r*^2^ correlation coefficients, all equation models, except for the zero-order model (0.799), fitted fairly well (0.992, 0.982, and 0.965 in the case of the first-order, Korsmeyer–Peppas, and Higuchi, respectively) showing the first-order kinetic model as the best fit to our experimental release data (Figure S6). This equation model, which assumes an exponential relationship between the amount of drug liberated and time, has exhibited the best fit in other types of drug delivery systems based on PLGA NPs [56]. Interestingly, both Korsmeyer–Peppas and Higuchi models also displayed good correlation coefficients (0.982 and 0.965, respectively) when fitted to our experimental data. We hypothesized that this good correlation might suggest a combination of two processes such as diffusion and erosion of the nanocarrier while RA is released through the polymeric network [57]. In addition, the presence of RA in the core of the nanoparticle might reduce the erosion effect of water molecules and consequently promote its sustained behaviour over time [53]. In this sense, the release of RA suggested a Fickian diffusion behaviour, according to Higuchi’s model (Figure S6). Additionally, the exponent *n*, which gives information on the release mechanism, was calculated from the Korsmeyer–Peppas equation (*n* = 0.48). This value of close to 0.5 suggests the presence of pores in the polymeric matrix that tend to be filled with the receptor solution during the diffusion process and favour bulk degradation. Therefore, this result seems to be consistent and supports an erosion–diffusion dependent mechanism for the release of RA, as observed in other PLGA polymeric NPs [53,58,59].

### 3.3. Protein Corona (PC)

Nanoparticles are prone to interact with a myriad of biomolecules present in the bloodstream when delivered in vivo. This process usually generates two layers of proteins that are adsorbed onto the NPs’ surface, namely hard and soft corona. The analysis and isolation of such adsorbed proteins have revealed that corona composition may change over time until reaching an equilibrium state [60] and is mainly composed of abundant proteins such as albumin and other less abundant ones that tend to interact selectively with the NPs’ surface [41,61]. Interestingly, the presence of this corona layer may produce significant changes in their biological activity affecting the long-term fate of NPs. Consequently, a full understanding of the effect of such corona on the stability of NPs is essential for the appropriate design of nanotherapeutics. The formation of PC NPs between polymeric NPs (e.g., PLGA and PLGA-PEG) with plasma proteins (FBS), their biophysical characterization, and isolation have been reported [60,62,63,64,65,66]. In these studies, NPs were mainly prepared via solvent emulsification–diffusion methods [66] using PVA as a stabilizer together with the use of high-energy methods (e.g., ultracentrifugation or high-speed homogenizer).

The adsorption process of serum proteins, as well as other kind of model proteins onto the surface of PLGA NPs prepared by nano-emulsion templates and low-energy methods, have been recently reported. These studies evaluated the colloidal stabilities of such PC NPs in the presence of different serum concentrations [64,65] as well the formation, isolation, and analysis of proteins adsorbed on the surface of different functionalized polymeric NPs using high-resolution mass spectrometry (HR-MS) [41,62,63,66]. In that article, PLGA polymeric NPs were incubated in the presence of two fetal bovine serum (FBS) concentrations (5 and 25%) at 37 °C. A full characterization of the resultant PC_NPs in terms of ζ-potential, DLS, and PDI was obtained after 24 h incubation. Unexpectedly, we observed that the stability and homogeneity of our series of NPs were compromised leading to nanoparticle aggregation and high PDI values of our final formulations. Further improvements in the colloidal stability of these PC NPs involving the surface decoration with poly(ethylene glycol) (PEG) might reduce this undesirable aggregation and be a potential strategy for intravenous administration.

Bearing in mind these precedent results, unloaded PLGA and RA-loaded PLGA NPs were incubated in the presence of 10% FBS to simulate in vitro cell culture conditions for 1 and 5 h at 37 °C. These incubations favoured proteins to be adsorbed on the NP surface leading to well-dispersed NPs at both incubation times (Figure S7). The average size, surface charge, and PDI of the resultant PC_NPs (PLGA_PC and RA-loaded PLGA_PC) were determined (Table 2 and Figure S8) as well as the concentration of adsorbed proteins which was quantified using the BCA assay (Figure S9) [67]. As expected, we observed that the resultant PC_PLGA NPs afforded larger average sizes when compared to their unmodified NP counterparts at both incubation times. Thus, the average diameter of the PC nanoconstructs was 94.2 and 95.3 nm for PLGA_PC and 129.6 and 137.3 nm for RA-loaded PLGA_PC after 1 and 5 h incubation, respectively [68].

**Figure 3:**
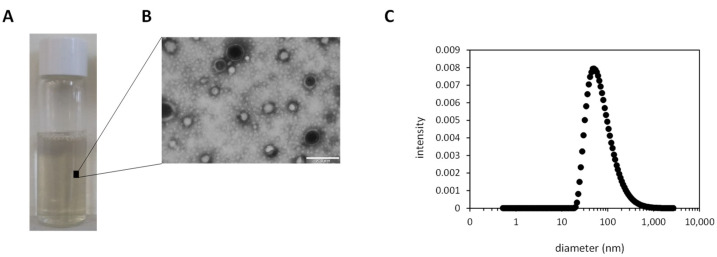
(**A**): Physical appearance of RA-loaded PLGA_PC NPs after 1 h incubation. PLGA polymeric NPs were centrifuged and resuspended in 1× PBS; (**B**): TEM microscopy image of RA-loaded PLGA_PC NPs showing the spherical morphology of PC NPs. Scale bar = 200 nm; (**C**): DLS size distribution of RA-loaded PLGA_PC NPs after 1 h incubation.

Interestingly, the presence of proteins adsorbed on the NP surface did not produce a negative effect on the polydispersity of PLGA_PC NPs (PDI values ranging from 0.17 to 0.22). Figure 3A shows the visual aspect of an RA-loaded PLGA_PC formulation after 1 h incubation. The TEM micrograph image (Figure 3B) confirms the spherical morphology of the NPs with sizes in agreement with the size distribution obtained by DLS (Figure 3C). We also noticed that protein concentration slightly increased as the incubation time was enlarged in the case of PLGA_PC NPs (103.1 and 141.82 µg·mL^−1^ for 1 and 5 h incubation, respectively) [68,69,70]. Surprisingly, this trend was not observed for RA-loaded PLGA_PC NPs in which the corona quantification went from 188.1 to 118.7 µg·mL^−1^ at both incubation times. Furthermore, the presence of the corona in both polymeric NPs significantly increased their ζ-potential values after 1 and 5 h incubation if compared with the corresponding control experiments (PLGA and RA-loaded PLGA NPs) [71].

Curiously, ζ-potential values did not change significantly in the case of non-loaded PLGA_PC NPs (−25.5 and −25.1 mV for 1 and 5 h incubation, respectively) [64]. Contrary to PLGA_PC NPs, ζ-potential was reduced to −20.4 mV in the case of RA-loaded PLGA_PC NPs after long incubation times presumably due to the replacement of proteins with short residence times and lower affinities by those with higher affinities [72,73].

The adsorption process between the major components of FBS (i.e., albumin) with the surface of polymeric NPs is a dynamic equilibrium. In addition to albumin, other proteins may also be part of the hard and soft corona which makes the hydrodynamic diameter of PLGA NPs increase. In the case of RA-loaded NPs, we hypothesized that RA, a polyphenol small molecule, might contribute to favouring electrostatic interactions with the major protein component of FBS, namely albumin [74]. Thus, the presence of RA, which may be located close to the surface of the drug-loaded NPs, might give rise to intermolecular π-π stacking between the aromatic rings of RA and the proximity of the corona containing aromatic amino acid chains to the NP surface [75,76]. Besides, additional non-covalent interactions of adsorbed albumin and NPs (non-loaded and RA-loaded) such as van der Waals forces and hydrogen bonding might co-exist affecting NPs' fate [37]. Interestingly, certain proteins might be desorbed as the incubation time is enlarged enriching the corona with the selective adsorption of less ubiquitous proteins on the NP surface [41,77]. This desorption process might be influenced by the presence of RA within polymeric NPs which might also contribute to additional interactions and vary the kinetics of binding of proteins leading to a slight decrease of the protein concentration at long-term incubation times.

Control experiments confirmed that unloaded PLGA NPs and RA-loaded PLGA NPs did not show any noticeable change in their diameter size and PDI in the absence of FBS when compared to incubated samples at 500 μg·mL^−1^. In this regard, PLGA NPs showed a diameter size of 41.8 nm and 43.4 nm for 1 and 5 h, respectively whereas the RA-loaded PLGA average diameter was 86.8 and 87.5 nm for the corresponding incubation times. As expected, none of these two control samples showed aggregation (i.e., particle sizes remained unchanged) after incubation at both temperatures (Figure S10).

### 3.4. In Vitro Antioxidative Activity and EC_50_

RA like other phytochemicals has shown promising therapeutic properties owing to its antioxidant activity and ability to protect mitochondria functionality [78]. This fact makes phytochemicals potential candidates for the treatment of certain neurodegenerative disorders [79].

**Figure 4:**
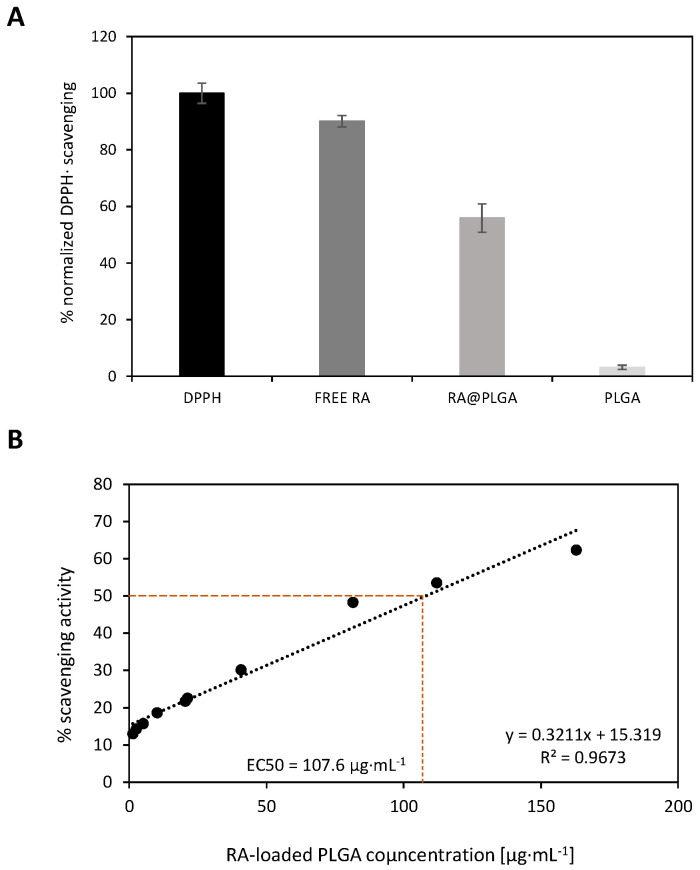
(**A**): Normalized antioxidant capacity of PLGA, RA-loaded PLGA, and free RA. The working concentration of bare, loaded PLGA, and free RA was 0.112 mg·mL^−1^. (**B**): Dose–response experiment involving the DPPH· scavenging activity versus RA-loaded PLGA concentration. To calculate the apparent EC_50_ value, a linear regression equation (y = 0.3211x + 15.319) (*r*^2^ = 0.9673) was obtained. Data are averaged over three independent experiments.

Although these antioxidant and free radical scavenging properties are widely known, they might vary when these small molecules are entrapped in NPs. We used an in vitro radical scavenging assay to evaluate the antioxidant activity of RA-loaded PLGA NPs using 2,2-diphenyl-1-picrylhydrazyl radical (DPPH·) [80]. This radical molecule, in the presence of antioxidant compounds, is able to accept a hydrogen or an electron leading to a change in color which quantitatively permits the measurement of their activity by absorbance at 517 nm [81]. Firstly, we selected a concentration of 0.112 mg/mL to evaluate the radical scavenging activities of RA-loaded PLGA NPs using both free RA and PLGA itself as positive and negative controls, respectively (Figure 4A).

The scavenging activity varied if RA was dissolved in an aqueous buffer or when entrapped within the polymeric NPs. As expected, the use of free RA displayed the greatest activity (90%) of the series whereas the antioxidant activity was reduced up to 56% in the case of RA-loaded PLGA at 0.112 mg/mL. This reduction in the scavenging activity might also corroborate the presence of RA within the polymeric network whereas its antioxidant activity was also preserved. Non-loaded PLGA showed a null scavenging effect, as expected.

Additionally, the EC_50_ value (concentration required to get 50% of antioxidant activity) for RA-loaded PLGA (Figure 4B) and free RA was calculated (Figure S11A). In this sense, we found the scavenging activity of RA-loaded PLGA increased in a dose-dependent manner ranging from 13% to 62% at concentrations of 1.27 to 163 µg·mL^−1^, respectively. To determine the apparent EC_50_ value, a linear regression equation (Y = 0.3211x + 15.319) with a correlation value of 0.96 was used to fit the data [82] affording a mean value of 107.6 ± 6.97 µg·mL^−1^ (Figure 4B). As expected, a lower EC_50_ value was obtained (5.34 µg·mL^−1^) [83] for RA in solution using a similar linear regression analysis that produced the equation Y = 7.4328x + 10.297 (*r*^2^ = 0.98) (Figure S11B).

### 3.5. Cytotoxicity

**Figure 5:**
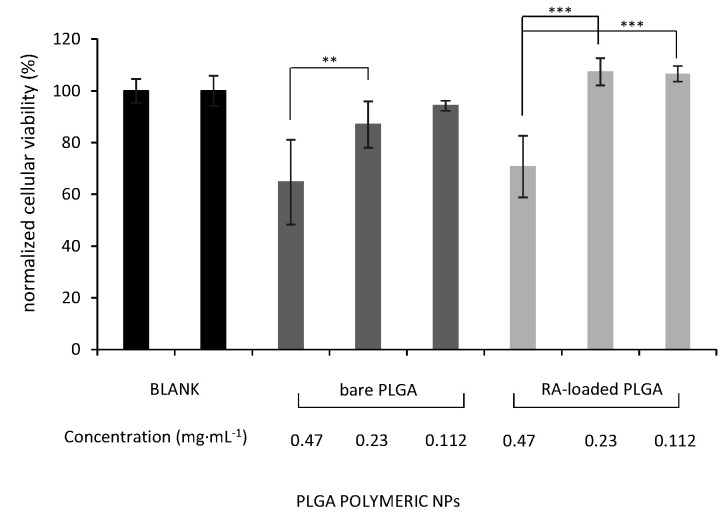
Cellular viability of SH-SY5Y cells upon treatment with increasing concentrations of PLGA polymeric NPs: non-loaded (bare) and RA-loaded PLGA NPs at 37 °C for 24 h. Cellular viability corresponds to the ratio between non-treated and treated cells. The first blank corresponds to untreated cells and the second one corresponds to the incubation of untreated cells with PBS. Data are average ± SD of three independent experiments (** *p* < 0.005 and *** *p* < 0.001).

The effect of unloaded and loaded PLGA NPs on cellular viability was evaluated using the MTT colourimetric assay [84]. Three PLGA concentrations of PLGA and RA-loaded PLGA (0.47, 0.23, and 0.112 mg·mL^−1^) were studied on SH-SY5Y as a model neuronal cell. Surprisingly, not all concentrations of PLGA were appropriate to be used in cell culture exhibiting significant cytotoxicity (** *p* < 0.005) at high concentration (0.47 mg·mL^−1^) after 24-h incubation in our conditions (65 and 71% for PLGA and RA-loaded PLGA NPs, respectively). On the contrary, these unexpected outcomes obtained at high concentrations were not observed if the incubation time was only limited to 4 h using the same neuronal cell line [85]. One of the possible explanations might be the amount of residual ethyl acetate present in the polymeric NPs after preparation. However, this might have a negligible impact as the organic solvent was reduced in vacuo and polymeric NPs were further purified by dialysis. Similarly, other unlikely scenarios such as the ζ-potential and PC contributions can be ruled out [86,87]. To this end, additional in vitro tests would be required to get insight into this unexpected behaviour. More diluted concentrations (0.23, and 0.112 mg·mL^−1^) did not affect the cell's mitochondrial functionality. In this sense, unloaded polymeric NPs exhibited cellular viabilities of 87 and 94%, respectively whereas RA-loaded PLGA NPs did not show any toxicity in cell culture at both selected concentrations (100%) (*** *p* < 0.001) (Figure 5).

### 3.6. Cellular Uptake

**Figure 6:**
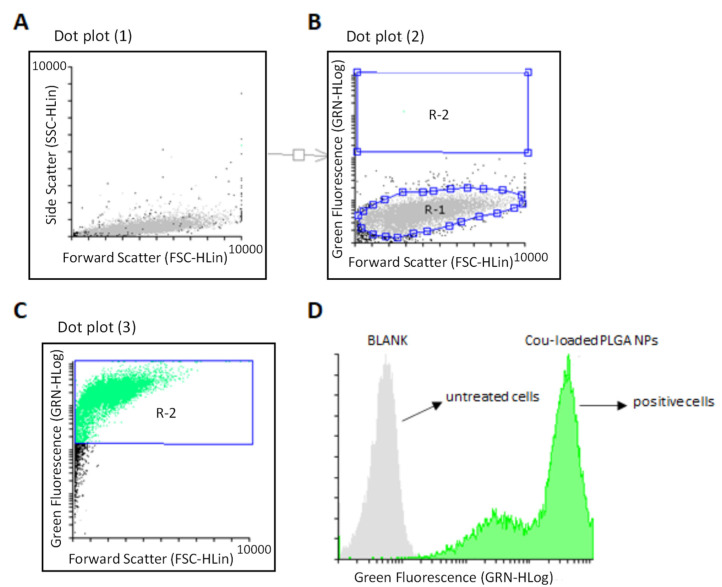
Flow cytometry analysis of Cou-labeled PLGA NPs in SH-SY5Y neuronal cells. (**A**): Normal cell population; (**B**): Selection of R1 and R2 gates for analysis; (**C**): Flow cytometry analysis for Cou-labeled PLGA NPs; (**D**): A flow cytometry histogram displays a shift among untreated cells and cell population containing Cou-labeled PLGA NPs. Transfection experiments were analyzed after 24 h incubation. A PLGA concentration of 0.23 mg·mL^−1^ was used together with 0.01 mg·mL^−1^ of Cou-6.

The ability of PLGA polymeric NPs to impart cellular uptake was investigated by flow cytometry. Fluorescently labelled NPs containing coumarin (Cou-6) (Cou-loaded PLGA) were prepared using the same emulsification method we have described above. We selected a Cou concentration (0.01 mg·mL^−1^) which was entrapped leading to stable PLGA colloidal dispersions with appropriate average size (57.9 nm) and good polydispersity values (0.3) (Figure S12). Gate regions R1 and R2 were defined with respect to side scatter differences [88] and were labelled with colours (grey and green, respectively). This selection allowed us to compare both non-labelled and fluorescent-labelled cell populations) in a scatterplot (forward scatter/side scatter dot plot) (Figure 6A,B). The analysis of the second gate (R2) confirmed that polymeric NPs loaded with Cou were principally taken by SH-SY5Y human neuroblastoma cells with uniform distribution (Figure 6C). These outcomes were also observed in a histogram which showed a remarkable shift between positive and non-fluorescence (Figure 6D). This may suggest that the integrity and stability of polymeric NPs were unaltered during the transfection process without causing any cellular perturbations during the internalization process.

## 4. Conclusions

PLGA NPs containing RA were prepared from nano-emulsion templates. Biophysical characterization of RA-loaded PLGA confirmed the entrapment of RA. In this sense, average NP sizes increased when compared with non-loaded PLGA NPs whereas ζ-potential showed larger absolute values than their unloaded PLGA NPs counterparts. This may suggest that RA remains close to the surface of NPs. The release of RA from NPs was studied in vitro at 37 °C. RA showed a sustained release profile reaching a plateau after ca. 5 h. Several kinetic models including zero-order, first-order, Higuchi, and Korsmeyer–Peppas were used to determine the best fit for our experimental data. According to *r*^2^ values, the first-order equation showed the best fit (*r*^2^ = 0.992) but Higuchi and Korsmeyer–Peppas also displayed good *r*^2^ values (0.965 and 0.982, respectively). Furthermore, the effect of the protein corona (PC) on the stability of non-loaded and RA-loaded PLGA NPs was studied at two incubation times (1 and 5 h). Interestingly, proteins adsorbed on the surface of NPs did not produce any negative effects on the colloidal stability of polymeric NPs at both incubation times. TEM results confirmed the spherical morphology of RA-loaded PLGA_PC nanoconstructs whereas average sizes were larger than their NP counterparts. Curiously, ζ potential values did not show significant changes and remained nearly constant after 1- and 5-h incubations. Additionally, antioxidant activities were studied using the DPPH· assay and confirmed radical scavenging activities (56%) at 0.112 mg·mL^−1^ with an EC_50_ value of 1.96 ± 4.79 µg·mL^−1^. Bare and RA-loaded PLGA NPs were incubated in the presence of SH-SY5Y neuronal cells. Cytotoxicity was measured at three PLGA concentrations showing significant toxicity at 0.47 mg·mL^−1^ in the case of non-loaded PLGA NPs whereas RA-loaded PLGA NPs did not affect the cellular proliferation process at any of the concentrations studied. Finally, cellular internalization studies using fluorescently labelled PLGA NPs confirmed their ability to impart efficient cellular uptake. In summary, these results are a new contribution to the field of NEs and may shed some light on the future engineering of multifunctional polymeric NPs for the treatment of neurological disorders.

## Figures and Tables

**Table 1:** Biophysical characterization of PLGA and RA@PLGA polymeric NPs ^1^.

Entry	Nanoparticle (NP)	RA (mg)	Organic (Oil) Phase (EtOAc:EtOH)	Method ^2^	EE% ^3^	Average Diameter ^4^ (nm)	PDI	ζ-Potential ^5^ (mV)
1	PLGA	0	100:0	A	-	46.6 ± 0.68	0.29	−18.0 ± 6.4
2	RA-loaded PLGA	1.2	100:0	B	92.2 ± 4.62	74.7 ± 0.17	0.30	−21.9 ± 1.26
3	RA-loaded PLGA	1.6	100:0	B	80.7 ± 7.15	70.9 ± 2.31	0.29	−36.8 ± 1.96
4	RA-loaded PLGA	1.6	90:10	A	64.5 ± 4.80	167.9 ± 3.25	0.21	−35.5 ± 6.0

^1^ All values correspond to a 4 mL total volume of polymeric NPs with a PLGA concentration of 2.8 mg·mL^−1^. DLS measurements were carried out in 1× PBS (200 μL) whereas ζ-potential measurements were obtained diluting 20 μL of polymeric NPs in 980 μL of Milli-Q water. ^2^ Method: (A) vortex and sonication; (B) vortex. ^3^ Encapsulation efficiency (EE%) was obtained using reversed-phase high-performance liquid chromatography (RP-HPLC) interpolating intensity values from a calibration curve with linearity over RA concentration range (1–1000 µg·mL^−1^) (see Supplementary Materials). ^4,5^ DLS and ζ-potential values were obtained from triplicate samples.

**Table 2:** Biophysical characterization of the pre-formed protein corona (PC) adsorbed on the surface of PLGA polymeric NPs ^1^.

Sample (NPs)	Incubation Time (Hours)	FBS (10%)	Average Diameter (nm)	PDI	ζ-Potential (mV)	Protein Corona Concentration (µg·mL^−1^)
PLGA ^2^	1	No	41.8 ± 1.25	0.27	−12.7 ± 0.62	-
PLGA ^2^	5	No	43.4 ± 0.30	0.40	−13.1 ± 0.83	-
PLGA_PC	1	Yes	94.2 ± 2.49	0.22	−25.5 ± 4.73	103.1 ± 6.05
PLGA_PC	5	Yes	95.3 ± 4.45	0.21	−25.1 ± 3.61	141.8 ± 11.4
RA-loaded PLGA ^3^	1	No	86.8 ± 1.02	0.28	−19.0 ± 1.27	-
RA-loaded PLGA ^3^	5	No	87.5 ± 1.49	0.27	−13.1 ± 0.69	-
RA-loaded PLGA_PC	1	Yes	129.6 ± 4.85	0.18	−33.0 ± 2.65	188.1 ± 1.75
RA-loaded PLGA_PC	5	Yes	137.3 ± 4.76	0.17	−20.4 ± 1.38	118.7 ± 8.33

^1^ Incubations were carried out using a PLGA concentration of 500 µg·mL^−1^ and 10% FBS at 37 °C to simulate in vitro cell culture conditions for 1 and 5 h. PC nanoconstructs were ultracentrifuged to remove unbound proteins and were resuspended in 1× PBS (2.0 mL). ^2,3^ NPs were used as controls at 500 µg·mL^−1.^

## Supplementary Materials

The following are available online at https://www.mdpi.com/article/10.3390/ma15134572/s1. Figure S1: Chemical structure of Rosmarinic acid (RA) and RA-loaded PLGA NPs; Figure S2: DLS size distribution of unloaded PLGA NPs; Figure S3: DLS size distibutions of RA-loaded PLGA NPs; Figure S4: Dark-field microscopy studies; Figure S5: Calibration curve of RA; Figure S6: Fitted curve release kinetic models; Figure S7: PLGA_PC images; Figure S8: DLS size distributions of PLGA_PC and RA-loaded PLGA_PC; Figure S9: Calibration curve of a model protein; Figure S10: PLGA NPs images in the absence of 10%FBS; Figure S11: In vitro scavenging effect of free RA and EC50 estimation; Figure S12: DLS size distribution of fluorescently labelled polymeric NPs; Table S1: HPLC conditions; Table S2: Drug release parameters.
