# Supplementary material for: Rosmarinic Acid-Loaded Polymeric Nanoparticles Prepared by Low-Energy Nano-Emulsion Templating: Formulation, Biophysical Characterization, and In Vitro Studies
Source: Materials (Basel). 2022 Jun 29;15(13):4572. doi: 10.3390/ma15134572 (PMC9267406; doi:10.3390/ma15134572)
Supplement: Supplementary file 1 [file materials-15-04572-s001.zip › materials-1778228-supplementary.pdf]

Supporting Information

Rosmarinic acid-loaded polymeric nanoparticles prepared by low-energy nano-emulsion templating: formulation, biophysical characterisation, and *in vitro* studies

Jessica García-Melero, Joan-Josep López-Mitjavila, María José García-Celma, Carlos Rodriguez-Abreu and Santiago Grijalvo

CONTENT

Figure S1. Chemical structure of Rosmarinic acid (RA) and RA-loaded PLGA NPs 2

Table S1. HPLC conditions 2

Figure S2. DLS size distribution of unloaded PLGA NPs 3

Figure S3. DLS size distibutions of RA-loaded PLGA NPs. 3

Figure S4. Dark-field microscopy studies 4

Figure S5. Calibration curve of RA 5

Figure S6. Fitted curve release kinetic models 5

Table S2. Drug release parameters 6

Figure S7. PLGA_PC images 6

Figure S8. DLS size distributions of PLGA_PC and RA-loaded PLGA_PC 7

Figure S9. Calibration curve of a model protein 7

Figure S10. PLGA NPs images in the absence of 10%FBS 8

Figure S11. *In vitro* scavenging effect of free RA and EC_{50} estimation 8

Figure S12. DLS size distribution of fluorescently labelled polymeric NPs 9

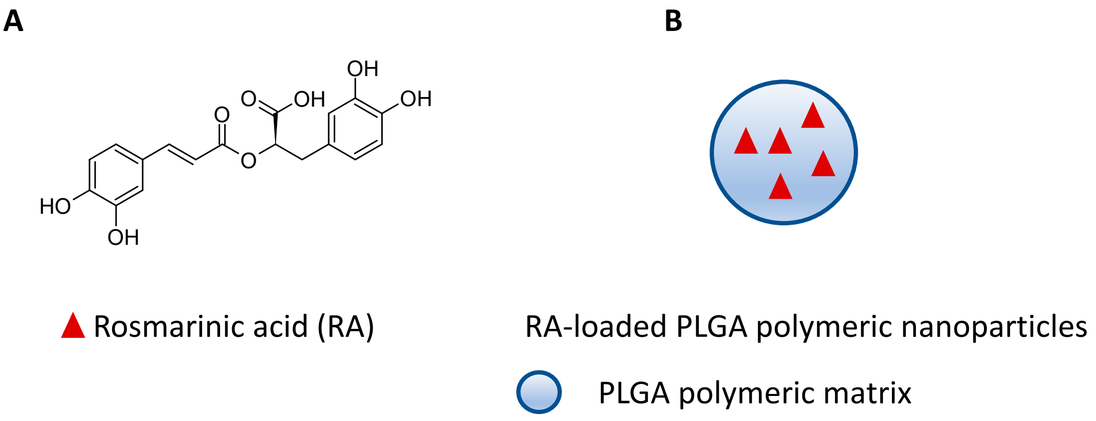

Figure S1. A. Chemical structure of Rosmarinic acid (RA); B. Representation of RA-loaded polymeric nanoparticles (NPs) used in this article

Table S1. Gradient elution conditions used to analyze RA elution by HPLC with a flow rate of 1 mL·min^{-1}.

Time (min)	%Water (A)	%Acetonitrile (B)
0	100	0
5	90	10
10	70	30
15	50	50
16	0	100
18	50	50
20	100	0

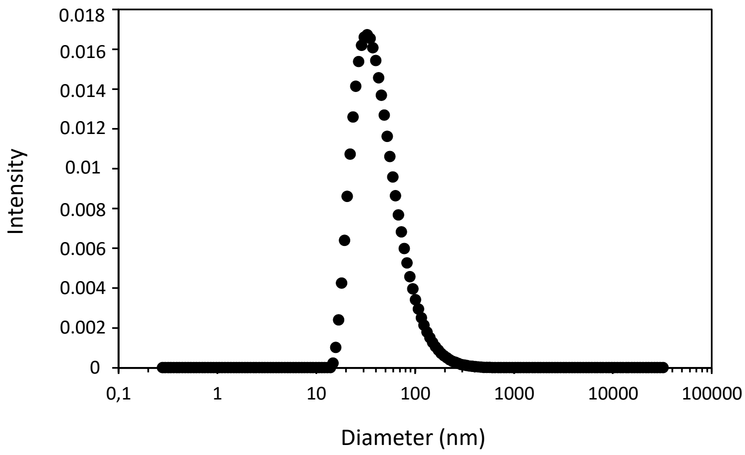

Figure S2. DLS size distribution of unloaded PLGA NPs

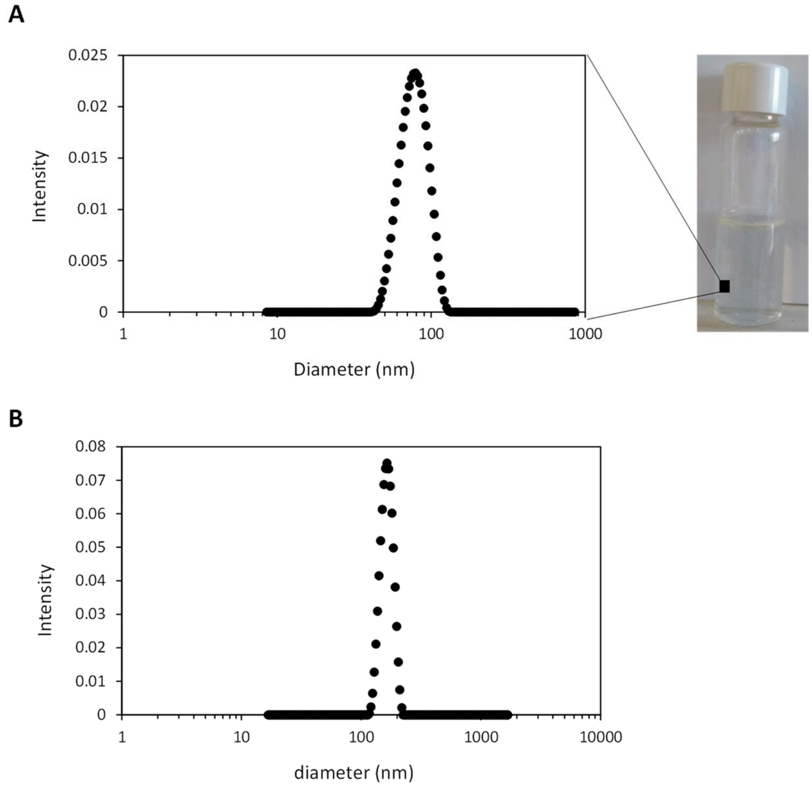

Figure S3. DLS size distributions of RA-loaded PLGA NPs prepared from ethyl acetate (A) and a mixture of ethyl acetate and ethanol in a ratio of 90:10 (B), respectively.

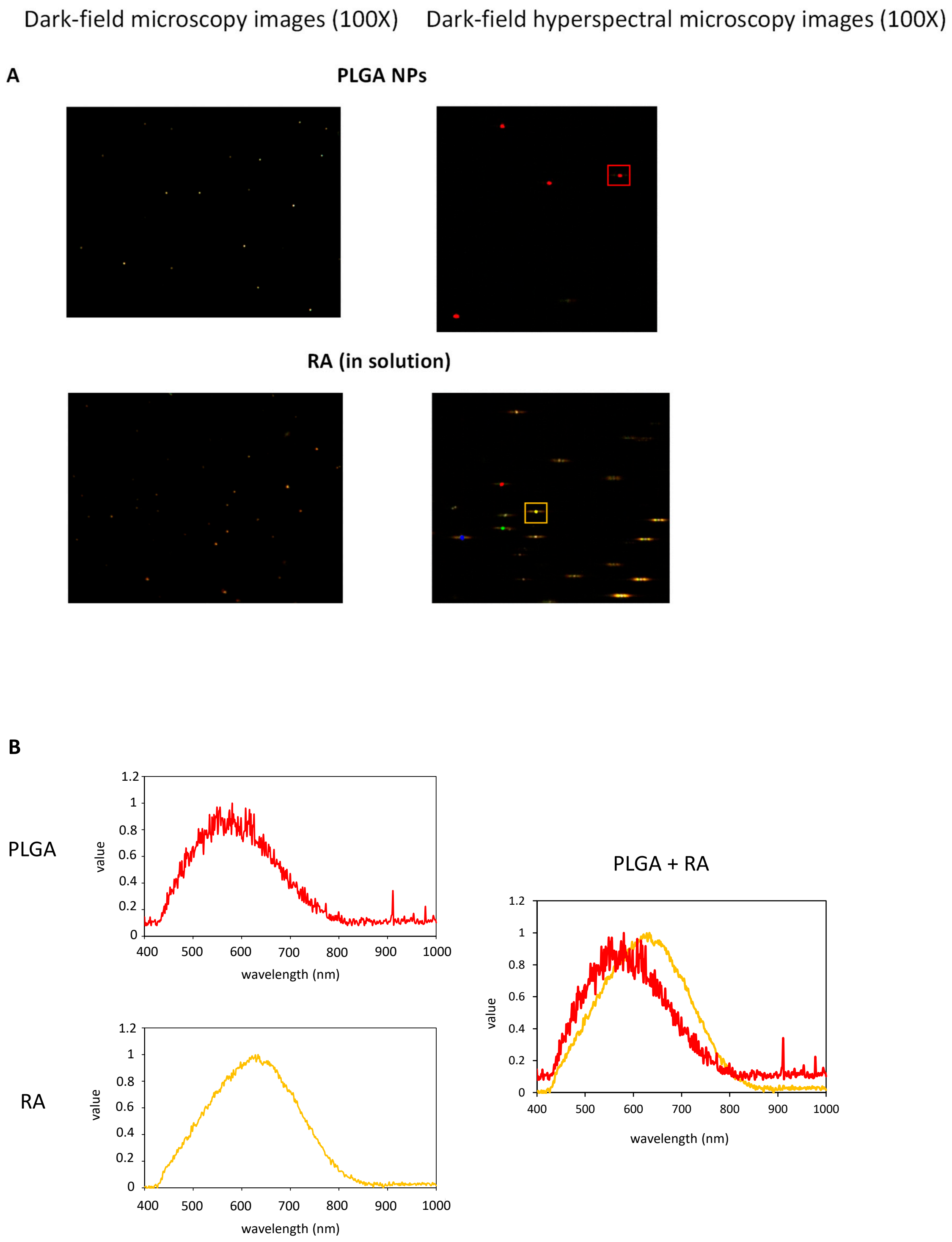

Figure S4. A. Dark-field and dark-field hyperspectral microscopy images of PLGA NPs and RA; B. Scattering spectra (normalized to the lamp spectrum) of a selected region of PLGA NPs and RA (see insert)

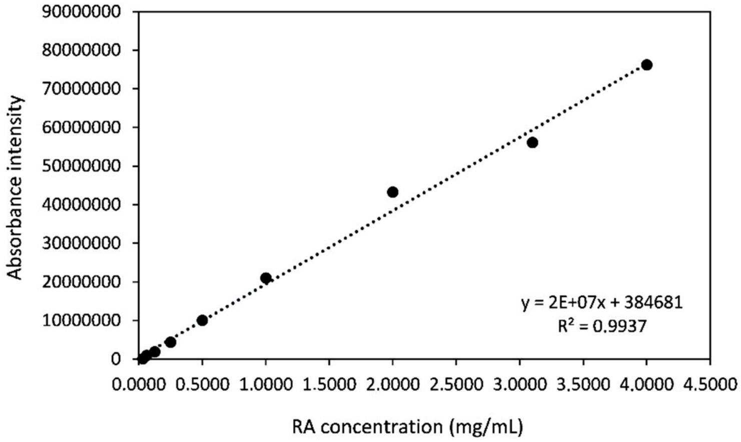

Figure S5. HPLC calibration curve for RA

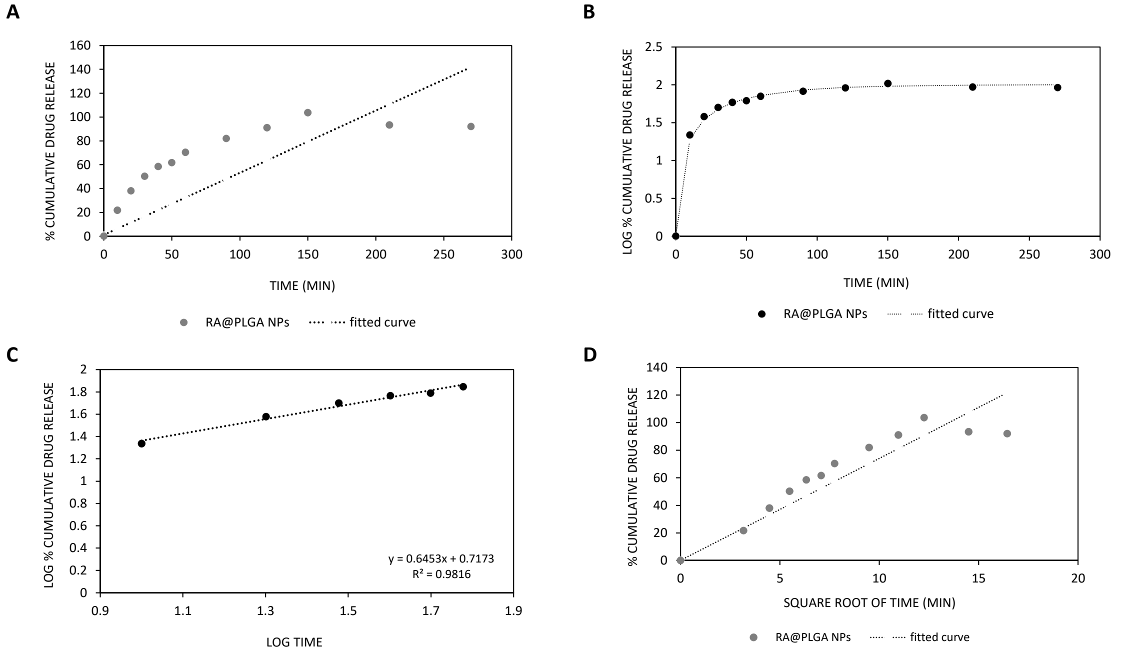

Figure S6. Fitted curves for RA release experimental data using (A) zero-order; (B) first-order; (C) Korsmeyer-Peppas, and (D) Higuchi equation kinetic models. For D, data were fitted for the 60% of the drug release from the nanoparticles.

Table S2. Drug release parameters for RA-loaded PLGA NPs according to zero-order, first-order, Korsmeyer, and Higuchi equation models

Zero-order		First-order		Korsmeyer-Peppas			Higuchi	
$\frac{M_t}{M_\infty} = Q_0 \times K_0 \times \mathrm{t}$		$\frac{M_t}{M_\infty} = 100 \times (1 - e^{-K*t})$		$\frac{M_t}{M_\infty} = K_{K-P} \times t^n$			$\frac{M_t}{M_\infty} = K_H \times \sqrt{t}$	
K_0	r^2	K	r^2	K_{K-P}	n	r^2	K_H	r^2
0.52	0.799	0.021	0.992	9.24	0.48	0.982*	7.40	0.965

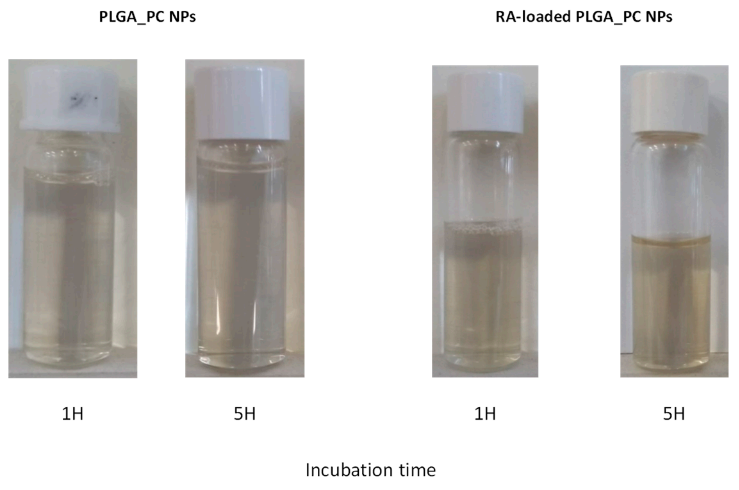

Figure S7. Visual aspect of PLGA_PC NPs (non-loaded and RA-loaded) at two incubation times (1 and 5 hours) at 37 °C and 10% FBS.

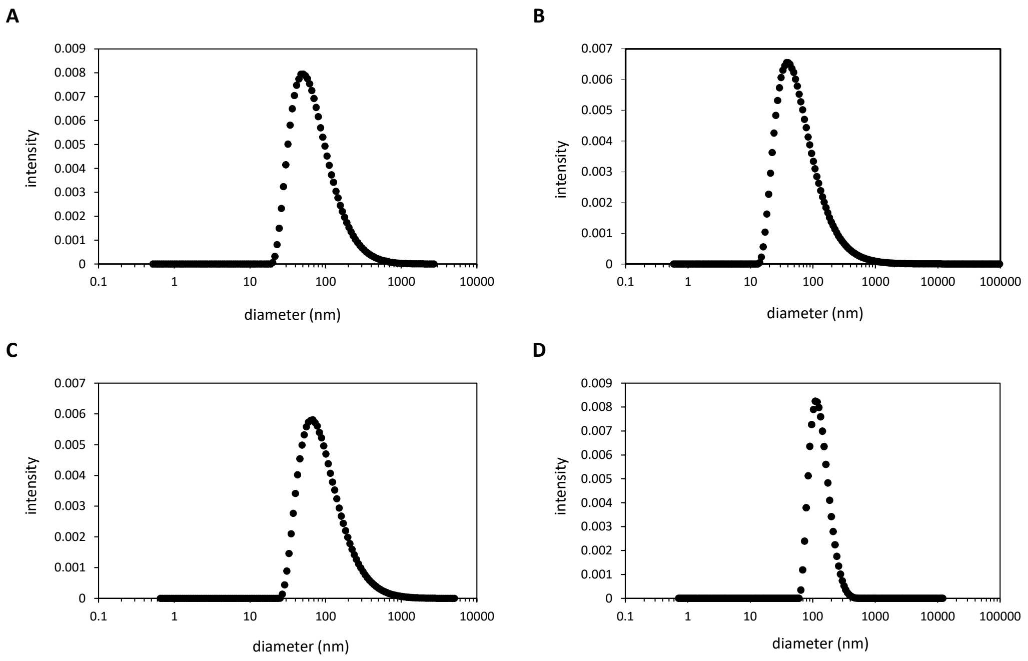

Figure S8. DLS size distributions of PLGA_PC (A, B) and RA-loaded PLGA_PC (C, D) after 1 (A, C) and 5-hour (B, D) incubation. PC NPs were isolated by ultracentrifugation and resuspended in 2 mL of 1X PBS

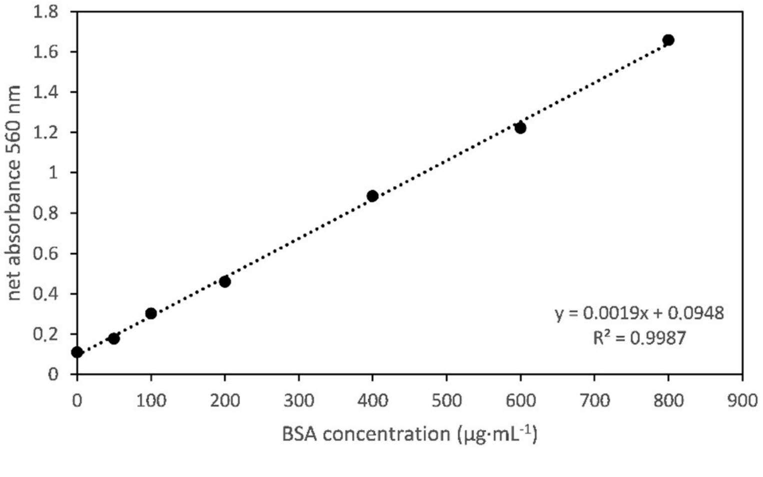

Figure S9. Calibration curve using a model protein (BSA) at different concentrations ranging from 100 to 800 µg·mL^{-1}. The equation Y=0.0019x+0,0948 was used to quantify the protein corona concentration adsorbed onto the surface of PLGA NPs

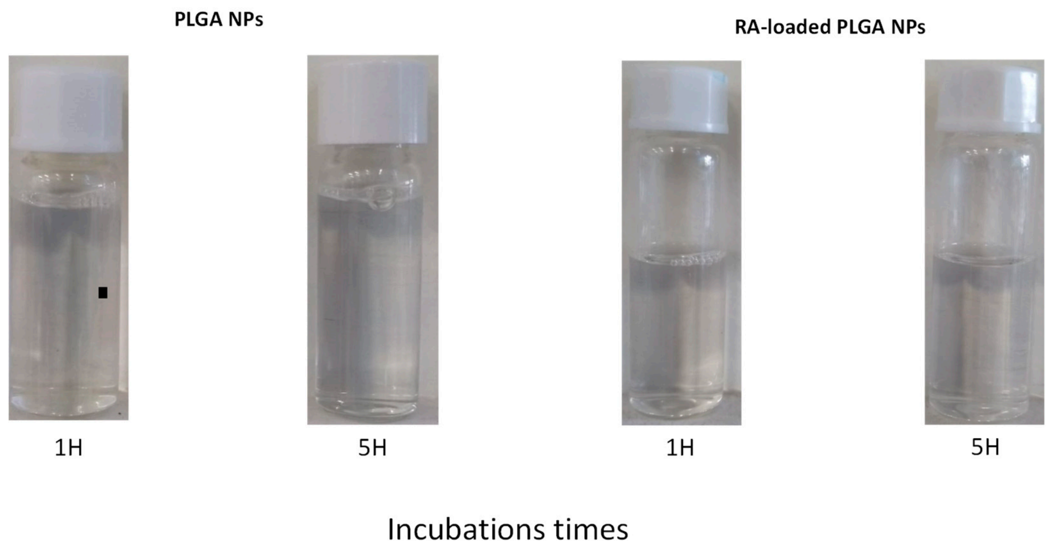

Figure S10 Visual aspect of PLGA NPs (non-loaded and RA-loaded) at two incubation times (1 and 5 hours) at 37 ^{o}C in the absence of 10% FBS.

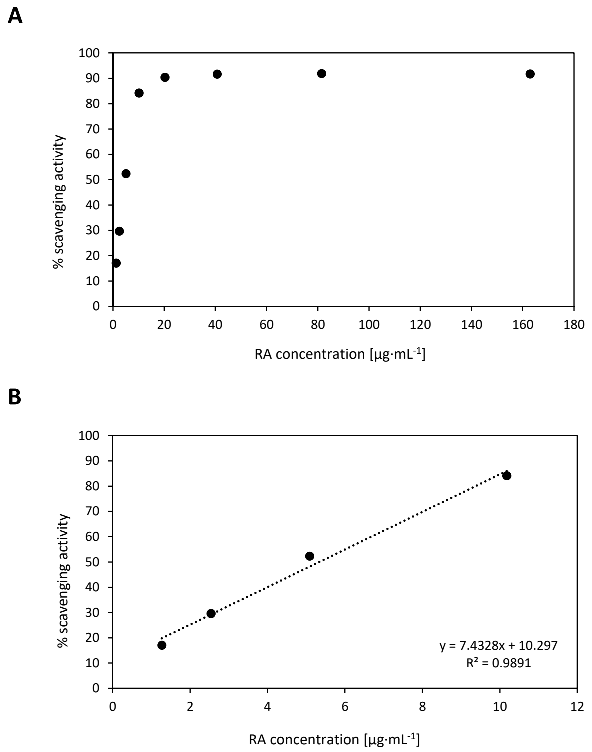

Figure S11. *In vitro* scavenging effect of free RA using DPPH· assay (A) and EC_{50} estimation calculated from a regression line equation (B)

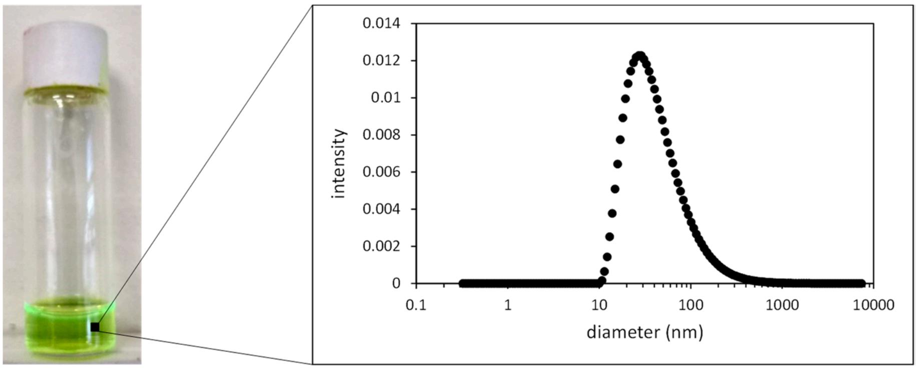

Figure S12. DLS size distribution of fluorescently labelled polymeric NPs containing coumarin-6
